# Brain MRI imaging characteristics predict treatment response and outcome in patients with *de novo* brain metastasis of EGFR-mutated NSCLC

**DOI:** 10.1097/MD.0000000000016766

**Published:** 2019-08-16

**Authors:** Chia-Ying Lin, Chao-Chun Chang, Po-Lan Su, Chien-Chung Lin, Yau-Lin Tseng, Wu-Chou Su, Yi-Ting Yen

**Affiliations:** a Department of Medical Imaging; b Division of Thoracic Surgery, Department of Surgery, National Cheng Kung University Hospital, College of Medical College, National Cheng Kung University; c Department of Internal Medicine; d Department of Internal Medicine and Institute of Clinical Medicine, National Cheng Kung University Hospital, College of Medicine, National Cheng Kung University; e Division of Trauma and Acute Care Surgery, Department of Surgery, National Cheng Kung University Hospital, College of Medical College, National Cheng Kung University, Tainan, Taiwan.

**Keywords:** brain metastasis, EGFR-tyrosine kinase inhibitors (TKIs), non-small-cell lung cancer

## Abstract

Patients with non-small cell lung cancer (NSCLC) and de novo brain metastasis (BM) have poor prognosis. We aim to investigate the characteristic of brain magnetic resonance (MR) imaging and the association with the treatment response of epidermal growth factor receptor-tyrosine kinase inhibitors (EGFR-TKIs) for lung cancer with BM.

EGFR-mutated NSCLC patients with BM from October 2013 to December 2017 in a tertiary referral center were retrospectively analyzed. Patient's age, sex, cell type, EGFR mutation status, treatment, and characteristics of BM were collected. Survival analysis was performed using Kaplan–Meier method. The efficacy of different EGFR-TKIs were also analyzed.

Among the 257 eligible patients, 144 patients with Exon 19 deletion or Exon 21 L858R were included for analysis. The erlotinib group had the best progression free survival (PFS) (median PFS 13 months, *P* = .04). The overall survival (OS) revealed no significant difference between three EGFR-TKI groups. Brain MR imaging features including tumor necrosis, rim enhancement and specific tumor locations (frontal lobe, putamen or cerebellum) were factors associated with poor prognosis. Patients with poor prognostic imaging features, the high-risk group, who received erlotinib had the best PFS (median PFS 12 months, *P* < .001). However, the OS revealed no significant difference between 3 EGFR-TKI groups. The low risk group patients had similar PFS and OS treated with three different EGFR-TKIs.

In NSCLC patients with common EGFR mutation and de novo BM, those with poor prognostic brain MR characteristics, erlotinib provided better PFS than afatinib or gefitinib.

## Abstract 2

Supplemental Digital Content is available in the text

## 1 Introduction

Lung cancer is the most common origin of metastatic brain tumor in adult patients.^[1]^ About 10% of the patients have brain metastasis (BM) on the diagnosis of lung cancer, and about 40% of patients developed BM during the treatment course.^[2]^ Patients with BM have poor prognosis and poor quality of life. About 30% of the patients with BM respond to chemotherapy, and combined radiotherapy and chemotherapy have been reported to improve survival.^[3]^ The blood-brain barrier has been regarded as the major hurdle of chemotherapeutic agent penetration. Whole brain radiotherapy (WBRT), stereotactic radiotherapy (SRT) and surgical resection serve as treatment for local control in symptomatic patients.

The development of EGFR-TKIs has shed light on the treatment of NSCLC with BM. Target therapy for molecularly selected NSCLC patients has been proven effective with acceptable toxicity for both intracranial and systemic disease simultaneously.^[4]^ Publications have shown that EGFR-TKIs have better intracranial efficacy than chemotherapy in terms of overall response rate, disease control rate, median progression fress survival (PFS), and median overall survival (OS).^[5,6,7,8]^ The MR imaging has been regarded as the standard diagnostic modality for brain lesions.^[9]^ Although it has been reported that the pattern and distribution of BM were associated with NSCLC mutation status, the association with treatment response and survival has not been investigated.^[10,11,12,13,14]^ Moreover, the real-world experiences and comparison of different TKI on EGFR-mutated NSCLC with de novo BM need to be stratified and analyzed. In this study we delineated the brain MR imaging characteristics and their association with prognosis and treatment outcome of different EGFR-TKIs.

## 2 Materials and methods

### 2.1 Patient population

The retrospective study was approved by our institutional review board (A-ER-107–316). A total of 257 patients diagnosed as lung cancer with BM between October 2013 and December 2017 in a tertiary referral center were reviewed. All the diagnoses were pathologically confirmed on the primary tumor using transthoracic needle biopsy or bronchoscopic biopsy, or on the surgical specimen of brain metastases. EGFR mutation test was conducted in each patient. Patients with primary lung adenocarcinoma and EGFR mutation were included and stratified according to the mutation type.

We recorded the baseline characteristics of the patients, including age, sex, histopathology cell type, EGFR mutation subtypes, intracranial and extracranial metastasis, and performance status. All the patients took gefitinib, erlotinib, or afatinib as the first line treatment at the discretion of the healthcare providers on disease diagnosis. Treatment modalities following EGFR-TKIs were also recorded. Disease progression was determined based on the radiographic evidence according to Response Evaluation Criteria in Solid Tumors (RECIST) version 1.1.

### 2.2 Acquisition of brain MR imaging

All brain MR examinations were performed with a 1.5T or 3T MR scanners within our institution, Achieva 1.5T (Philips Healthcare, Best, the Netherlands) MR scanner, 1.5T (GE Healthcare, Signa HDxt) MR scanner, or 3T (Ingenia, Philips Healthcare, Best, the Netherlands) MR scanner.

The protocols of MR imaging were as the following: axial spin echo T1-weighted imaging (T1WI), fast spin-echo T2-weighted imaging (T2WI), fluid attenuated inversion recovery (FLAIR), T2∗-weighted gradient-recalled echo (GRE) or SWI (3D GRE) images. The DWI was performed by applying sequentially in the x, y, and z direction, and ADC maps were obtained from these imaging data. Contrast-enhanced (CE) images obtained in axial, coronal, sagittal T1WI and axial 3D T1 fast-spoiled gradient-recalled imaging after intravenous administration of 0.2 mmol/kg of body weight of gadolinium-based contrast agent. Detailed imaging parameters in the MR scanners can be found in Supplementary file 1.

### 2.3 Evaluation of brain MR imaging

**Figure 1:**
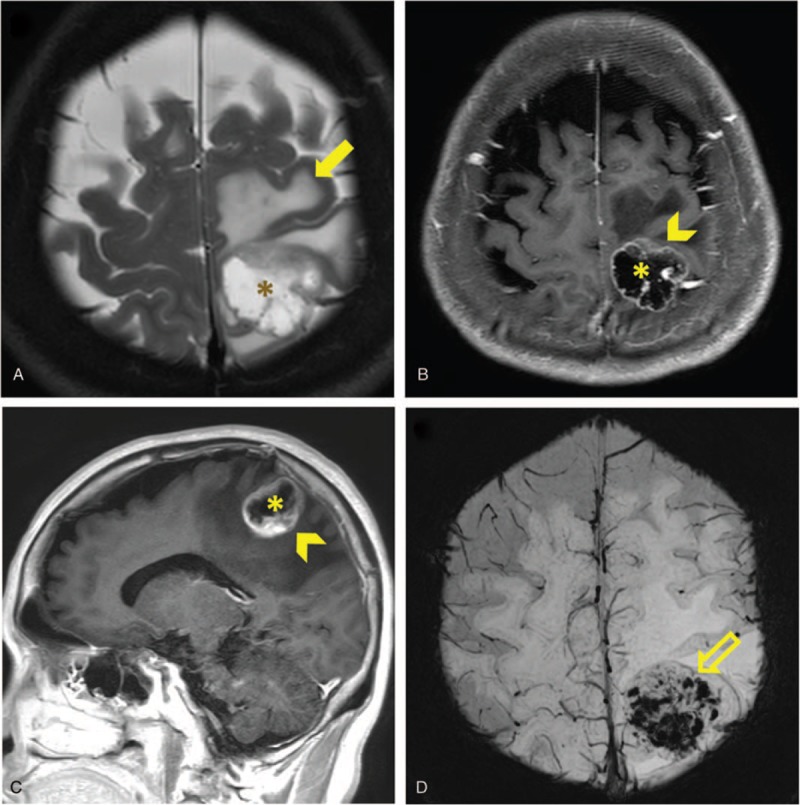
Representative MR images showing tumor necrosis, rim enhancement, peri-tumoral edema, and hemorrhage. A 66-year-old female NSCLC patient with left parietal metastasis. Axial nonenhanced T2-weighted MR images (A) shows central necrosis (asterisk) and peri-tumoral vasogenic edema (arrow). Axial (B) and sagittal (C) contrast-enhanced T1-weighted MR image shows central necrosis (asterisk) and rim enhancement (arrowhead). Axial susceptibility weighted image (D) shows hypointensities within the tumor (hollow arrow), suggesting hemorrhage.

The MR imaging was retrospectively analyzed by a broad certificated neuroradiologist (C.Y.L.) blinded to the clinical and pathologic information. The tumor location, number, maximum diameter of the largest lesion, presence of tumor necrosis, rim enhancement, peri-tumoral edema, or hemorrhage (Fig. 1) were evaluated as MR morphologic features. The brain tumor location was divided into ten areas, including frontal lobe, parietal lobe, temporal lobe, occipital lobe, caudate nucleus, putamen, thalamus, insula, cerebellum, and brainstem. The maximum diameter of the largest lesion was measured based on axial view. The presence of tumor necrosis was determined by hyperintensity on T2WI imaging. The presence of peri-tumoral edema was detected on axial T2WI and FLAIR imaging. The presence of hemorrhage was detected on GRE or SWI imaging.

### 2.4 EGFR mutation analysis of lung cancer

The tissue of primary or metastatic lung cancer was obtained for EGFR mutation analysis. Tissue sample consisting over 80% tumor content, as determined via microscopy with hematoxylin and eosin staining, were selected for the study. The QIAcube automated extractor (Qiagen, Hilden, Germany) with the QIAamp DNA FFPE tissue kit (Qiagen) eluted in ATE (QIAmp Tissue Elution) buffer (Qiagen) were used to extract DNA according to the manufacturer's instructions. The presence of EGFR mutations was determined using the EGFR PCR Kit (EGFR RUO Kit) and therascreen EGFR RGQ PCR Kit (EGFR IVD Kit). These kits combined Scorpions and the amplification-refractory mutation system (ARMS) technologies to detect the mutations using real-time quantitative PCR.

### 2.5 Statistical analysis

Chi-square or Fisher exact test was used to compare the categorical variables, and independent *t* test or ANOVA was used to evaluate the continuous variables of patient's characteristics. Estimations of PFS and OS were made with the Kaplan–Meier method, and Cox proportional hazards regression was used to determine factors associated with PFS and OS. PFS and OS were defined as time interval from the commencement of EGFR TKI treatment to documented disease progression or death from any cause. The patients with poor prognostic factors were defined as high risk group. A *P* value of ≤.05 was set to indicate statistical significance. SPSS system (IBM SPSS Statistics, Version 22.0) was used for statistical analysis.

## 3 Results

### 3.1 Demographic and clinical findings

**Figure 2:**
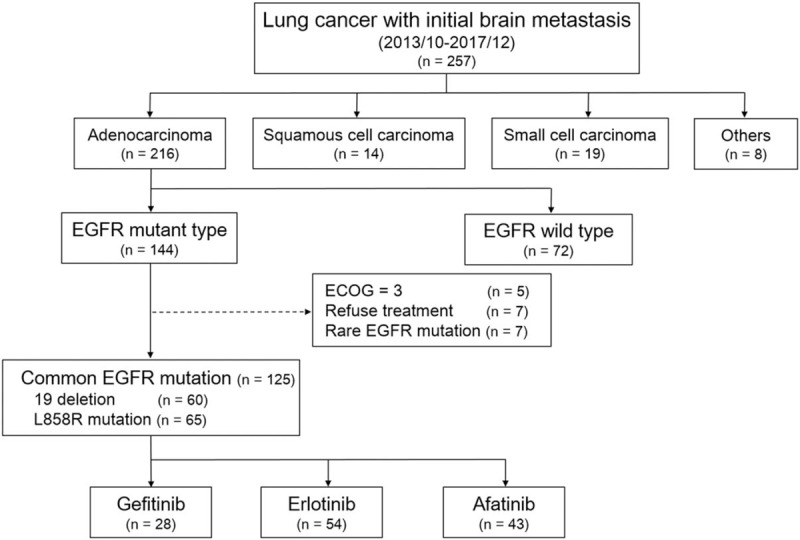
Flow diaphragm of patient selection.

From October 2013 to December 2017, a total of 257 patients were diagnosed as lung cancer with de novo BM. Of these patients, 216 patients had adenocarcinoma, and 144 (56.3%) of them were documented to have EGFR mutation. Patients who had poor performance status, that is, Eastern Cooperative Oncology Group (ECOG) ≥3 (n = 5), who refused further treatment (n = 7), and whose EGFR mutation status were other than 19 deletion or L858R mutation (n = 7) were excluded. Eventually, 125 patients were included in this study. Among these patients, 60 patients had exon 19 deletions and 65 patients had exon 21 L858R mutations (Fig. 2). Of the included patients, 28 patients were given gefitinib, 54 patients erlotinib, and 43 patients afatinib as the first line therapy. The demographic data and brain MR imaging features are summarized in Table 1. The afatinib group had the largest proportion of female patients, younger patients, patients with better performance status (ECOG PS < 2), and patients undergoing chemotherapy, although the difference was not significant. There was no statistical difference in brain MR imaging tumor characteristics, tumor locations or other extracranial metastatic sites among three different EGFR-TKI groups.

**Table 1:**
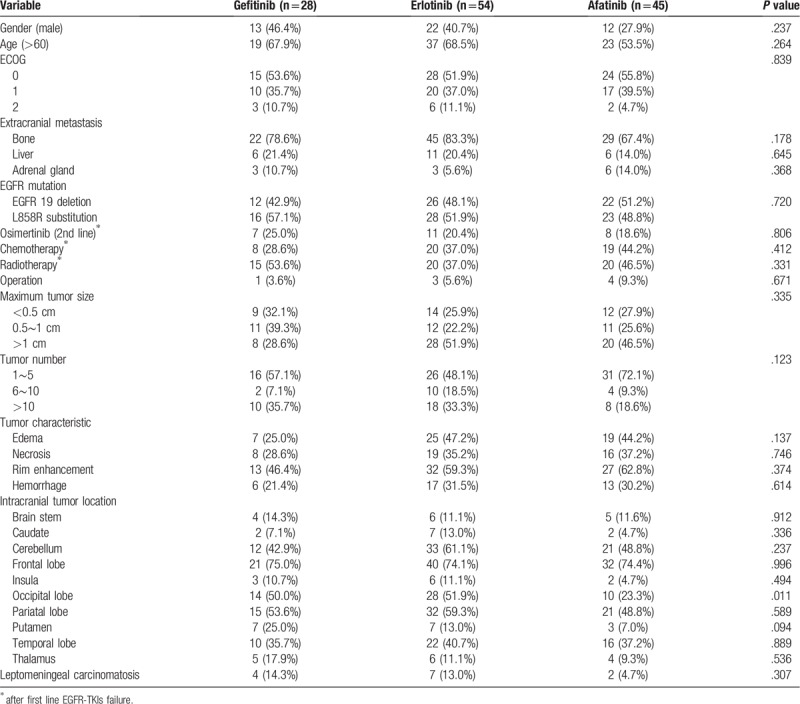
The demographic data and MR imaging features.

### 3.2 Prognostic factors of clinical and brain MR imaging characteristics

**Figure 3:**
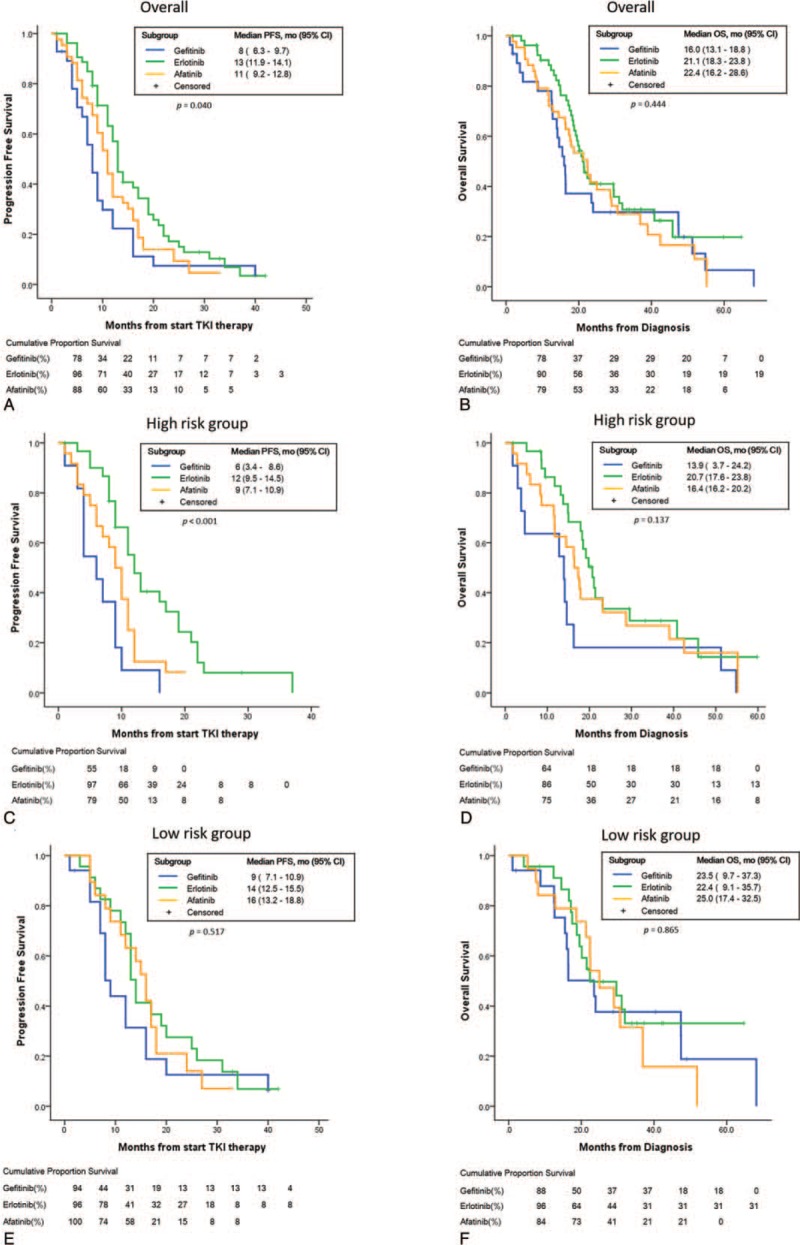
Kaplan–Meier survival curves verified by log-rank test. (A, B) Comparable PFS and OS in patients treated with gefitinib, erlotinib or afatinib. (C, D) In high risk cohorts, erlotinib showed better PFS but comparable OS to gefitinib or afatinib. (E, F) In low risk cohorts, erlotinib showed comparable PFS and OS to gefitinib or afatinib.

Tables 2 and 3 show the results of univariate and multivariate analyses of the clinical and brain MR imaging prognosticators of the PFS and OS. The erlotinib group had the best PFS (median PFS 13 months, 95% CI: 11.9–14.1; *P* = .04). The OS revealed no significant difference among three EGFR-TKI groups. (Fig. 3A and B)

**Table 2:**
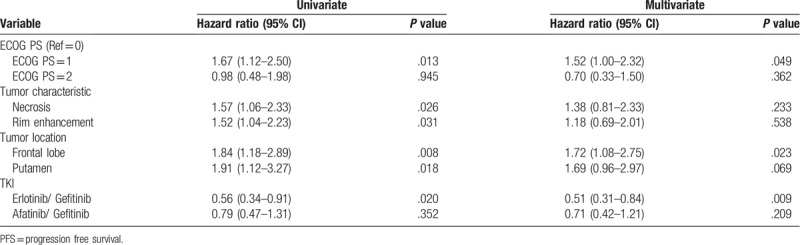
Univariate and multivariate cox proportional hazard analysis for PFS.

**Table 3:**
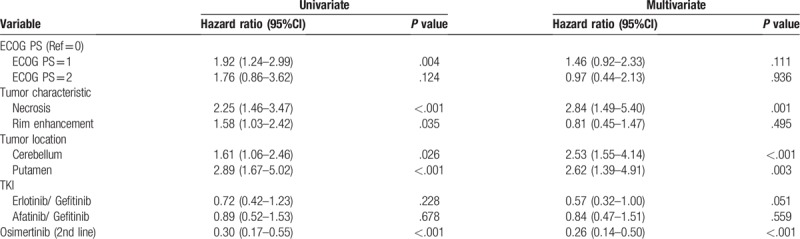
Univariate and multivariate cox proportional hazard analysis for overall survival.

The univariate analysis for prognosticators in PFS revealed that performance status (ECOG 1 vs 0, HR: 1.67, 95% CI: 1.12–2.50; *P* = .013), tumor characteristics as necrosis (HR: 1.57, 95% CI: 1.06–2.33; *P* = .026) or rim enhancement (HR: 1.52, 95% CI: 1.04–2.23; *P* = .031), tumor location at frontal lobe (HR: 1.84, 95% CI: 1.18–2.89; *P* = .008) or putamen (HR: 1.91, 95% CI: 1.12–3.27; *P* = .018). The multivariate analysis revealed that the performance status (ECOG 1 vs 0, HR: 1.52, 95%CI: 1.00–2.32; *P* = .049) and metastasis at frontal lobe (HR: 1.72, 95%CI: 1.08–2.75; *P* = .023) were associated with PFS.

The univariate analysis for OS revealed that performance status (HR: 1.92, 95%CI: 1.24–2.99; *P* = .004), tumor characteristics as necrosis (HR: 2.25, 95% CI: 1.46–3.47; *P* < .001) or rim enhancement (FR: 1.58, 95% CI: 1.03–2.42; *P* = .035), BM at cerebellum (HR: 1.61 95% CI: 1.06–2.46; *P* = .026) or putamen (HR: 2.89, 95% CI: 1.67–5.02; *P* < .001), and second line osimertinib administration (HR: 0.30, 95% CI: 0.17–0.55; *P* < .001) were associated OS. The multivariate analysis revealed that tumor characteristics as necrosis (HR: 2.84, 95%CI: 1.49–5.40; *P* = .001), BM at cerebellum (HR: 2.53 95% CI: 1.55–4.14; *P* < .001) or putamen (HR: 2.62, 95% CI: 1.39–4.91; *P* = .003), and second line osimertinib administration (HR: 0.26, 95% CI: 0.14–0.50; *P* < .001) were associated OS. The erlotinib group had marginally superior OS to the gefitinib group (HR 0.57, 95% CI: 0.32–1.00, *P* = .051).

### 3.3 PFS and OS of high-risk group patients

The patients with poor prognostic MR imaging features, including tumor necrosis, rim enhancement, and specific tumor locations (frontal lobe, putamen, and cerebellum), were defined as high risk group. Accordingly, we compared the treatment response of three different EGFR-TKIs (erlotinib, afatinib, and gefitinib).

In high risk group, patients treated with erlotinib had a better PFS than gefitinib or afatinib (median PFS 12 versus 6 or 9 months, *P* < .001) but similar OS (median survival: erlotinib, gefitinib versus afatinib = 20.7, 13.9 vs 16.4 months, *P* = .137), whereas low risk group patients had similar PFS (median survival: erlotinib, gefitinib versus afatinib = 14, 9, 16 months, *P* = .517) and OS (median survival: erlotinib, gefitinib vs afatinib = 22.4, 23.5 vs 25.0, *P* = .865) (Fig. 3C–F).

## 4 Discussion

To the best of our knowledge, this is the first study utilizing brain MRI characteristics as a prognostic factor and response predictor in patents with EGFR-mutated NSCLC treated with different EGFR-TKIs as the first line therapy. Our study results indicated that in patients with NSCLC of EGFR-sensitizing mutation with de novo BM, erlotinib provided better PFS than afatinib or gefitinib but comparable OS as afatinib or gefitinib if the patients had poor prognostic MR characteristics of BM, including tumor necrosis, rim enhancement and specific tumor locations (frontal lobe, putamen and cerebellum). After first line EGFR-TKI failure, the OS was longer in patients with T790M-mutant NSCLC who underwent subsequent osimertinib administration. Therefore, in NSCLC patients with initial BM, subsequent treatment directed by driver gene mutation after first line EGFR-TKI failure might provide more therapeutic effect and survival benefit than conventional chemotherapy.

The previous studies have shown certain MR imaging characteristics were associated with gene mutation status of the primary tumor^[14]^ and were predictor for OS.^[15]^ However, these studies did not further focus on the association between brain MRI characteristics and prognosis, and had minimal impact on the treatment decision. There have been a few publications focusing on ADC value as brain MR parameters and its association with BM. DWI parameters, minimum ADC and normalized ADC ratio, for the solid BM was reported to predict the EGFR mutation status in BM from lung adenocarcinoma,^[14]^ and minimum ADC and ADC transition coefficient (ATC, ADC changes at the brain-metastasis interface) as predictor for OS.^[15]^ We found that the high prevalence of intratumoral hemorrhage or necrosis in BM is a major technical issue, and small BM was only detected on 3D T1 imaging and too small to be measured on ADC map. Therefore, we did not include ADC value as a brain MRI characteristic in the current study. There are limited data in the literature about the impact of brain MRI morphologic findings and enhancement patterns of the metastatic brain lesions on outcome. The real-world treatment experiences of EGFR-TKIs on brain metastatic NSCLC with common EGFR mutation have been reported, but few focusing on the neuroradiological appearance of BM and treatment efficacy. Brain tumors intersecting major white matter tracts such as the cortico-spinal tract, inferior fronto-occipital fasciculus, inferior longitudinal fasciculus, and anterior thalamic radiations are associated with decreased OS and PFS because of direct infiltration routes to the brain stem and other structures for vital physiological function.^[16]^ The prior studies showed that tumor location associated with different BBB permeability, which could result in various treatment outcome.^[17,18]^ The neuroradiologic appearance of tumor necrosis and rim enhancement is suggestive of neovascularization and rapid tumor growth, followed by lack of blood supply into the tumor and tissue hypoxia, resulting in reduced radiosensitivity and compromised penetration of therapeutic agents.^[19,20,21,22]^

Literature review of first and second generations EGFR-TKIs treatment in EGFR-mutated NSCLC with BM was summarized in Table 4. Recently, studies have revealed comparable OS and PFS among different EGFR-TKIs, gefitinib, erlotinib, and afatinib,^[23]^ but direct comparison between afatinib, gefitinib, and erlotinib as first-line therapies for advanced NSCLC with de novo BM is still lacking. It is believed that intracranial metastasis consists of brain parenchymal and leptomeningeal metastasis. Certain studies demonstrated that erlotinib showed better outcome than gefitinib in patients with BM patients with EGFR-sensitizing mutations.^[24,25]^ Preclinical and retrospective data showed that erlotinib provides better penetration rate in the central nervous system and objective responses in patients with BM from EGFR-mutated NSCLC than gefitinib or afatinib.^[4,26,27,28,29,30,31,32]^ Afatinib has also been documented to have substantial cerebrospinal fluid concentration because of its high affinity and irreversible binding as a second generation tyrosine-kinase inhibitor (TKI),^[30]^ and effective in patients with EGFR-mutated NSCLC with BM.^[33]^ The regression of CNS metastases observed during afatinib treatment has provided evidence that afatinib concentration in the CSF is sufficient to inhibit tumor growth due to its potency at relatively low concentrations.^[34]^ Notably, few of these studies investigated the efficacy of tyrosine-kinase inhibitor on patients with high-risk BM of EGFR-mutant advanced NSCLC. Small brain parenchymal metastasis might remain asymptomatic; leptomeningeal metastasis, the spread of malignant cells to the subarachnoid space within the compartment of the cerebrospinal fluid, often results in rapid deterioration of consciousness and performance status, and grave prognosis.^[35,36,37]^ Five people diagnosed with leptomeningeal metastasis were treated with erlotinib, and the proportion of patients undergoing radiotherapy for BM was marginally higher in the afatinib group. The presence of leptomeningeal metastasis in brain MRI imaging did not contribute negatively to the survival in the erlotinib group and radiotherapy did not contribute positively in the afatinib group. Our study demonstrated that in patients with high-risk metastatic brain lesions, erlotinib provided better progression-free survival but not OS than afatinib or gefitinib.

**Table 4:**
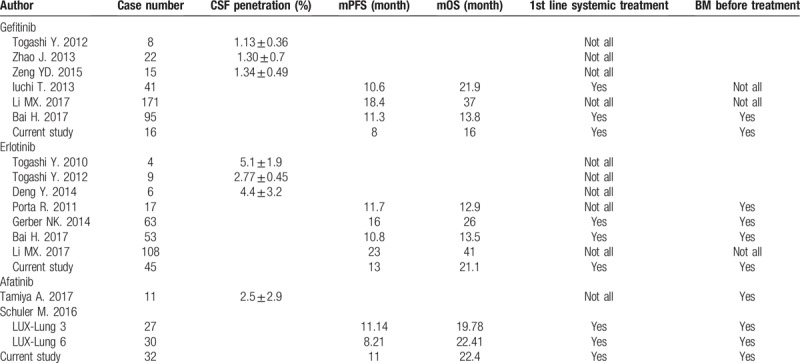
Literature review of first and second generations EGFR-TKIs treatment in EGFR-mutated NSCLC with brain metastasis.

Our study had limitations. First, it was a single center retrospective study with relatively small sample size and statistical power was therefore limited. Second, the choice among different EGFR-TKIs was based on the discretion of the healthcare providers, which could lead to selection bias. The site of progression, e.g. brain or other extracranial site, was not explicitly accounted for in our statistical analysis. In addition, after initial EGFR-TKIs treatment failure, rebiopsy to confirm the presence of the T790 M mutation is not routinely performed, thus not all patients took osimertinib (AZD9291) as second line therapy, which may potentially confound the results. Finally, the time of WBRT could influence the CNS EGFR-TKI concentration^[27]^ and has impact on PFS, however, there was only limited patients receiving WBRT, thus we did not further divide the patients into concurrent WBRT with EGFR-TKIs group and adjuvant WBRT after first line EGFR-TKIs failure. Future larger prospective studies are warranted to validate our study findings.

## 5 Conclusion

In selected patients with poor prognostic MR characteristics of BM, including tumor necrosis, rim enhancement and specific tumor locations (frontal lobe, putamen and cerebellum), erlotinib provided better PFS than afatinib or gefitinib.

## Author contributions

**Conceptualization:** Chia-Ying Lin, Chao-Chun Chang, Yau-Lin Tseng, Yi-Ting Yen.

**Data curation:** Chia-Ying Lin, Chao-Chun Chang, Po-Lan Su, Chien-Chung Lin, Wu-Chou Su, Yi-Ting Yen.

**Formal analysis:** Chia-Ying Lin, Chao-Chun Chang.

**Investigation:** Chia-Ying Lin, Chao-Chun Chang.

**Methodology:** Chia-Ying Lin, Chao-Chun Chang, Po-Lan Su, Chien-Chung Lin, Yi-Ting Yen.

**Resources:** Po-Lan Su.

**Supervision:** Chien-Chung Lin, Yau-Lin Tseng, Wu-Chou Su, Yi-Ting Yen.

**Writing – original draft:** Chia-Ying Lin, Chao-Chun Chang.

**Writing – review & editing:** Yi-Ting Yen.

Chia-Ying Lin orcid: 0000-0003-3248-2369.

## Supplementary Material

Supplemental Digital Content
