# Supplementary material for: Brain MRI imaging characteristics predict treatment response and outcome in patients with de novo brain metastasis of EGFR-mutated NSCLC
Source: Medicine (Baltimore). 2019 Aug 16;98(33):e16766. doi: 10.1097/MD.0000000000016766 (PMC6831109; doi:10.1097/MD.0000000000016766)
Supplement: Supplemental Digital Content [file medi-98-e16766-s001.docx]

**MRI Protocols**

The protocols of 1.5T (Philips Healthcare, Best, the Netherlands) MR imaging were as the following: axial T2WI (4927/120 ms; FOV, 20–23 cm; slice thickness/spacing, 5 mm/6 mm; matrix, 256 x 256), T2WI FLAIR (8000/140 ms; FOV, 20–23 cm; slice thickness/spacing, 5 mm/6 mm; matrix, 256 x 256), and T2-weighted GRE (393/14 ms; FOV, 23 cm; flip angle, 20°; slice thickness/spacing, 5 mm/6 mm; matrix, 256 x 224), axial T1-weighted spin-echo (T1WI) FLAIR (TR/TE, 2000/9 ms; FOV, 23 cm; slice thickness/spacing, 5 mm/6 mm; matrix, 256 x 256) , and MR angiography. Contrast-enhanced images obtained in axial, coronal, sagittal T1WI (2000/10ms; FOV, 20-23 cm; slice thickness/spacing, 5 mm/6 mm; matrix, 256 x 263), 3D T1WI (9/5 ms; FOV, 20-23 cm; slice thickness/spacing, 1 mm/1 mm; matrix, 197x232) were performed after intravenous administration of 0.2 mmol/kg of body weight of gadolinium-based contrast agent. The DWI was performed by applying sequentially in the x, y, and z directions with the following parameters: TR/TE, 5000/89 ms, flip angle, 90 degrees, slice thickness/spacing, 5 mm/6 mm; b = 0, and 1000 sec/mm2. ADC maps were obtained from these imaging data. ).

The protocols of 1.5T (GE Healthcare, Signa HDxt) MR imaging were as following: axial T2WI (6000/50 ms; FOV, 18.4-23 cm; slice thickness/spacing, 5 mm/6 mm; matrix, 320 x 192), T2WI FLAIR (9000/120 ms; FOV, 18.4-23 cm; slice thickness/spacing, 5 mm/6 mm; matrix, 256 x 224), T1WI FLAIR (TR/TE, 2250/9 ms; FOV, 23 cm; slice thickness/spacing, 5 mm/6 mm; matrix, 320 x 224), and T2-weighted GRE (650/15 ms; FOV, 23 cm; flip angle, 20°; slice thickness/ spacing, 5 mm/6 mm; matrix, 320 x 160), and MR angiography. Contrast-enhanced images obtained in axial, coronal and sagittal T1WI (2250/9 ms; FOV, 16-23 cm; slice thickness/spacing, 5 mm/6 mm; matrix, 320 x 224), 3D T1WI (10/4 ms; FOV, 19.2-24 cm; slice thickness/spacing, 1 mm/1 mm; matrix, 288 x 288) were performed after intravenous administration of 0.2 mmol/kg of body weight of gadolinium-based contrast agent. The DWI was performed by applying sequentially in the x, y, and z directions with the following parameters: TR/TE, 10000/87.8 ms, flip angle, 90 degrees, slice thickness/spacing, 5 mm/6 mm; b = 0, and 1000 sec/mm2. ADC maps were obtained from these imaging data. ).

The protocols of 3T (Ingenia, Philips Healthcare, Best, the Netherlands) MR imaging were as following: T2WI (4579/100 ms; FOV, 22-23 cm; slice thickness/spacing, 4 mm/5 mm; matrix, 328 x 246), T2WI FLAIR (11000/125 ms; FOV, 20-22 cm; slice thickness/spacing, 4 mm/5 mm; matrix, 308 x 224), axial T1WI (TR/TE, 2100/20 ms; FOV, 20-23 cm; slice thickness/spacing, 4 mm/5 mm; matrix, 328 x 229), and T2-weighted GRE (567/20 ms; FOV, 22-22 cm; slice thickness/ spacing, 5 mm/6.5 mm; matrix, 256 x 192). Contrast-enhanced axial, coronal, sagittal T1WI (2100/ 20 ms; FOV, 22-23 cm; slice thickness/spacing, 4 mm/5 mm; matrix, 3280 x 229), 3D T1WI (8/4 ms; FOV, 20-25.6 cm; slice thickness/spacing, 1 mm/1 mm; matrix, 256 x 200) after intravenous administration of 0.2 mmol/kg of body weight of gadolinium-based contrast agent. The DWI was performed by applying sequentially in the x, y, and z directions with the following parameters: TR/TE, 8000/64 ms; flip angle, 90 degrees, slice thickness/spacing, 5 mm/6 mm; b = 0 and 1000 sec/mm2. ADC maps were obtained from these imaging data.
